# The hidden arrow in the FedEx logo: Do we really unconsciously “see” it?

**DOI:** 10.1186/s41235-023-00494-x

**Published:** 2023-07-03

**Authors:** Shih-Chiang Ke, Ankit Gupta, Yu-Hui Lo, Chih-Chung Ting, Philip Tseng

**Affiliations:** 1 Graduate Institute of Mind, Brain and Consciousness, Taipei Medical University, Taipei, Taiwan; 2 Institute of Psychology, University of Hamburg, Hamburg, Germany; 3 Center for Research in Experimental Economics and Political Decision Making, University of Amsterdam, Amsterdam, Netherlands; 4 Cross College Elite Program, National Cheng Kung University, Tainan, Taiwan; 5 Department of Psychology, National Taiwan University, Taipei, Taiwan; 6 Psychiatric Research Center, Wan Fang Hospital, Taipei Medical University, Taipei, Taiwan; 7 Research Center for Mind, Brain & Learning, National Chengchi University, Taipei, Taiwan

**Keywords:** Implicit processing, Unconscious processing, Figure-ground segregation, Logo design, Consciousness, Biased competition model

## Abstract

The FedEx logo makes clever use of figure-ground ambiguity to create an “invisible” arrow in the background space between “E” and “x”. Most designers believe the hidden arrow can convey an unconscious impression of speed and precision about the FedEx brand, which may influence subsequent behavior. To test this assumption, we designed similar images with hidden arrows to serve as endogenous (but camouflaged) directional cues in a Posner’s orienting task, where a cueing effect would suggest subliminal processing of the hidden arrow. Overall, we observed no cue congruency effect, unless the arrow is explicitly highlighted (Experiment 4). However, there was a general effect of prior knowledge: when people were under pressure to suppress background information, those who knew about the arrow could do so faster in all congruence conditions (i.e., neutral, congruent, incongruent), although they fail to report seeing the arrow during the experiment. This was true in participants from North America who had heard of the FedEx arrow before (Experiment 1 & 3), and also in our Taiwanese sample who were just informed of such design (Experiment 2). These results can be well explained by the Biased Competition Model in figure-ground research, and together suggest: (1) people do not unconsciously perceive the FedEx arrow, at least not enough to exhibit a cueing effect in attention, but (2) knowing about the arrow can fundamentally change the way we visually process these negative-space logos in the future, making people react faster to images with negative space regardless of the hidden content.

The modern logo for Federal Express (FedEx) was designed by Lindon Leader in 1994, and has won numerous design awards worldwide. In 2003, the FedEx logo was lauded by Rolling Stone Magazine as one of the top 8 designs in three decades. The well-known secret to its success lies in the enclosed space between the uppercase ‘E’ and lowercase ‘x’, which creates a hidden white arrow that shares the same color as the background. In design, this clever use of the background is called “negative space”, and is popular among designers for its ability in adding meanings but without cluttering the design (Hardy, 2011). But most importantly, a popular assumption in design is that the hidden arrow should be picked up by the visual system implicitly, thereby creating the implicit impression of speed and precision for the FedEx brand (e.g., Atrees, 2015).

Is this assumption of people’s automatic and unconscious processing of the arrow really true? This is essentially a figure-ground segregation problem in vision science. That is, the design community assumes that the background (i.e., the arrow), or negative space, is unconsciously processed to a certain extent that is enough to alter subsequent higher-order cognitive processes. Evidence from perception literature seems to provide some support for both sides of the prediction. On one hand, competition for figural status can be resolved quickly (Qiu & von der Heydt, 2007), and the background not only becomes shapeless, but is suppressed (e.g., Cacciamani et al., 2015; Peterson & Skow, 2008). On the other hand, studies have also shown that object recognition can occur before (or at least in parallel with) figure-ground segregation (e.g., Peterson & Gibson, 1993; Vecera & O’Reilly, 1998). And importantly, even when the ground loses the competition for figural status, its semantic meaning can nonetheless be processed to induce a N400 component (Sanguinetti et al., 2014) and facilitate subsequent lexical decision-making (Cacciamani et al., 2014). Therefore, it may be that the white arrow is quickly suppressed as a shapeless background, or perhaps the directional cueing information is picked up before it loses the figure-ground competition—both of which are possible and thus the question about the FedEx arrow still remains an open question.

To investigate this, we designed 72 images with a similar hidden arrow (Fig. 1) and used it as an endogenous directional cue in a Posner’s orienting task. Previous research has already shown that arrows are powerful cues to orient attention (Posner, 1980). Therefore, if the hidden background arrow is indeed processed, its directionally-specific information should facilitate people’s reaction time towards the pointed side. Importantly, previous research in priming has raised the possibility of a moderating effect of prior knowledge (e.g., Yi, 1993). Therefore, we separated our participants into a “yes” (i.e., people who knew about the FedEx arrow) or “no” group (i.e., people who did not know) by asking whether they have previously heard about the hidden FedEx arrow (Online Study: Experiment 1, 3, and 4), or by experimentally manipulating their knowledge of the arrow (Lab Replication: Experiment 2).

**Fig. 1:**
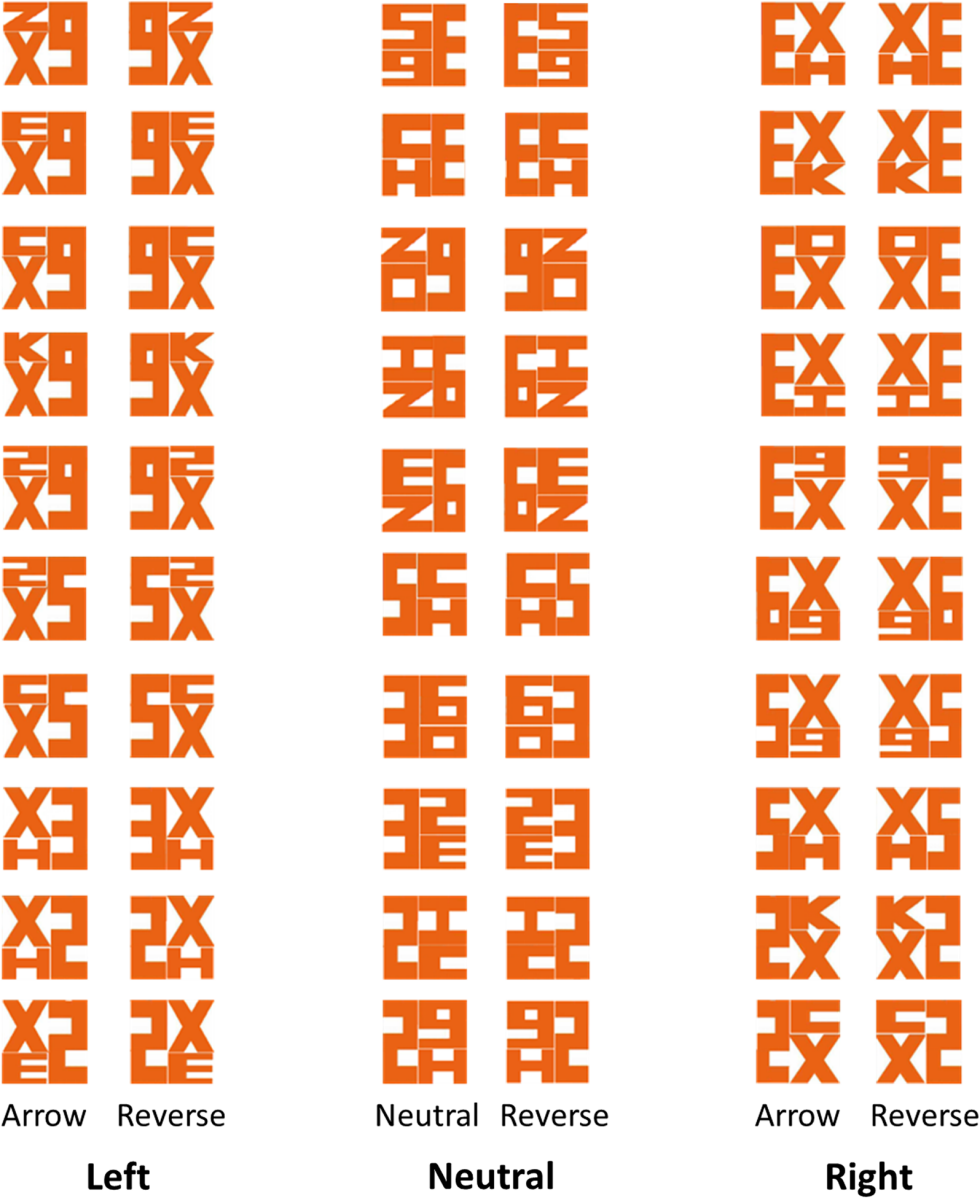
Table of stimuli that served as the central arrow cue in Experiment 1, 2, and 4. Hidden FedEx-like arrows were created by placing X either on the left or right side. The same alphanumeric combinations were used in reversed positions to create “reverse control” images such that X’s would appear equally often between left and right sides, with only half of the trials containing a hidden arrow. Reverse control images were also created for neutral images (middle column)

## Experiment 1: online study

In Experiment 1, to get a mixture of participants who may or may not have heard of the FedEx arrow, we recruited participants from North America using Amazon Mechanical Turk. Participants were not aware of the purpose of the experiment, and were only asked about their prior knowledge of the FedEx arrow at the very end of the experiment.

### Methods

#### Participants

One hundred and twenty-seven participants (72 male, 55 female, age 23~57, mean age = 36.62) from the United States participated via Amazon Mechanical Turk. The experiment was introduced as a speeded test of attention and memory, and nothing about the FedEx arrow was mentioned in the description. In the end, based on participants’ exit survey, we had 71 participants who knew about the FedEx arrow beforehand (“yes” group: 47 male, 24 female, age 23 ~ 56, mean age = 35.87) and 56 who did not (“no” group: 25 male, 31 female, age 24 ~ 57, mean age = 37.57). There was no significant age difference between these two groups [t_(125)_ = − 1.414, p = 0.160, Cohen’s d = − 0.253].

Eleven participants’ data were excluded from the analysis due to low accuracy in the two tasks (< 70%). Among them, 5 were excluded for accuracy < 70% in the cueing task, 3 for accuracy < 70% in the change detection task, and 3 were excluded for accuracy < 70% in both tasks. Six additional participants (3 from ‘yes’ group and 3 from ‘no’ group) were excluded because they reported noticing the arrows in the experimental stimuli while performing the experiment.

All participants gave informed consent via mouse click prior to their participation, and all received financial compensation for their time. All experimental procedures were reviewed and approved by the Joint Institutional Review Board of Taipei Medical University, Taiwan.

#### Stimuli

Participants performed an endogenous attentional cueing task. Instead of an explicit arrow, we replaced the central cue arrow with a FedEx-like figure. A total of 72 FedEx-like figures were created, 12 of which were used in the practice session, and the remaining 60 were used in the formal experiment (Fig. 1).

The FedEx-like figure is always composed of 3 alphanumeric symbols (i.e., x, E, H, c, K, o, I, z, 2, 3, 5, 6, 9), with either no hidden arrow (i.e., neutral trial), or an embedded white arrow that was either pointing left or right. To ensure physical similarity across all 72 images, the ratio between orange and white pixels were kept approximately at 3 to 1. The number of white and orange pixels were almost the same across all images (difference < 12%). Additionally, to make sure there was no obvious syntactical clues in the stimuli, only “X” was used to construct the pointy side of the arrow as it is the only letter that can create both left- and right-pointing arrows. An equal number of “reverse control” images were created so that X would appear equally often on the left and right side for both directional and neutral images (Fig. 1; 10 neutral/10 reverse control, 10 right/10 reverse control, 10 left/10 reverse control).

#### Task and procedure

**Fig. 2:**
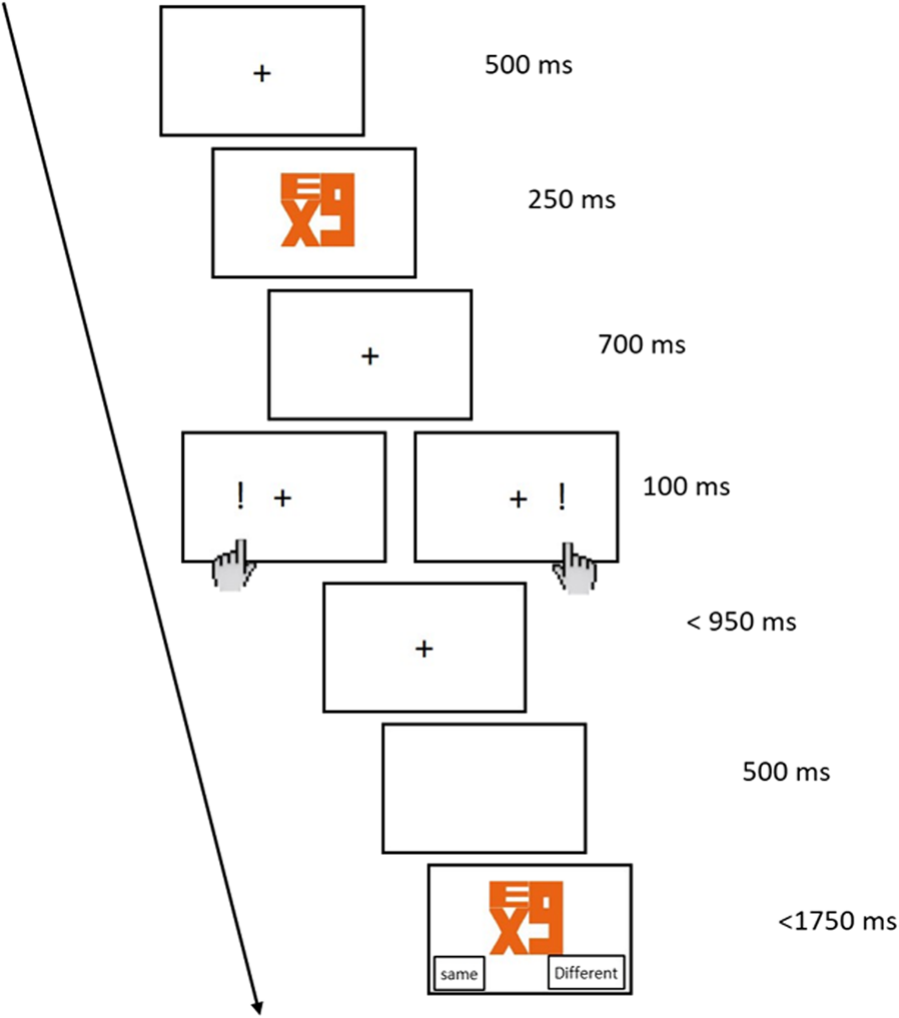
Procedure of the cueing task. Participants were to press the button according to the location of the target (i.e., exclamation mark), which can be congruent or incongruent with the cued direction. To ensure that participants were paying full attention to the cue, they were asked to perform a delay match-to-sample change detection task at the end of the trial

The entire experiment was programmed in JavaScript and hosted online. At the start of every trial, a 500 ms fixation cross was displayed at the center, followed by a neutral or directional symbol for 250 ms, a 700 ms fixation cross with a 100 ms target either on the left or right side, and another 950 ms fixation during which the participants had to respond with a left or right button press. After the participants’ response, the script would wait for 500 ms before a symbol would appear for 1750 ms, and the participants had to respond either this symbol was the same or different from the one they just saw in the beginning of the trial (Fig. 2). This post-trial match-to-sample, or change detection, task is designed to ensure the participants would pay full attention to the cue symbol. In the change detection task, whenever there is a change trial, the second image was randomly selected from the same pool of 60 images (Fig. 1). Participants were asked to react as fast as possible while being highly accurate to both the target location and the matching task.

The experiment consisted of a total of 160 trials, of which 80 were directional (40 congruent, 40 incongruent) and 80 were neutral. In the congruent condition, the direction of the hidden arrow would match the location of the target, and vice versa for the incongruent condition. Because the number of congruent and incongruent trials are the same (i.e., 40 and 40), the centrally-displayed arrow in this study is non-predictive of the actual target location, which has been shown by previous studies to still be able to induce shifts of endogenous attention, especially in the congruent condition, when the arrow is visible (Doricchi et al., 2010; Hommel et al., 2001). All trial orders were randomized across all participants. Participants on average took 20 min to complete the entire task.

#### Post-experiment questions

Since our experiment did not mention anything related to design or the FedEx arrow, to gauge the participants’ prior knowledge of the FedEx arrow, participants were asked 4 questions (in the same order) after they had completed the task and before they exit the experiment: (1) What did you think was the real purpose of the experiment? (2) Did you notice anything in the symbols in this experiment? (3) Did you notice a white arrow in some of the symbols in this experiment? (4) Before participating in this experiment, did you know about the hidden arrow in the FedEx logo? (accompanied by an image of FedEx logo).

### Results and discussion

We first analyzed accuracy to verify participants’ attentiveness in the online experiment. The accuracy of the cueing task and the trial-end change detection from all participants was 98.90% and 94.71%, respectively. Based on people’s prior knowledge of the FedEx arrow, the respective accuracy for the ‘yes’ group was 98.87% and 95.03%, and for the ‘no’ group it was 98.95% and 94.31%. There was no statistically significant difference between the two groups in accuracy [Cueing**: **t_(125)_ =  − 0.291, p = 0.771, Cohen’s d = − 0.052; Change detection: t_(125)_ =  − 1.113, p = 0.268, Cohen’s d = − 0.199]. Therefore, all participants were attentive to the FedEx-like images and performed the task well.

**Fig. 3:**
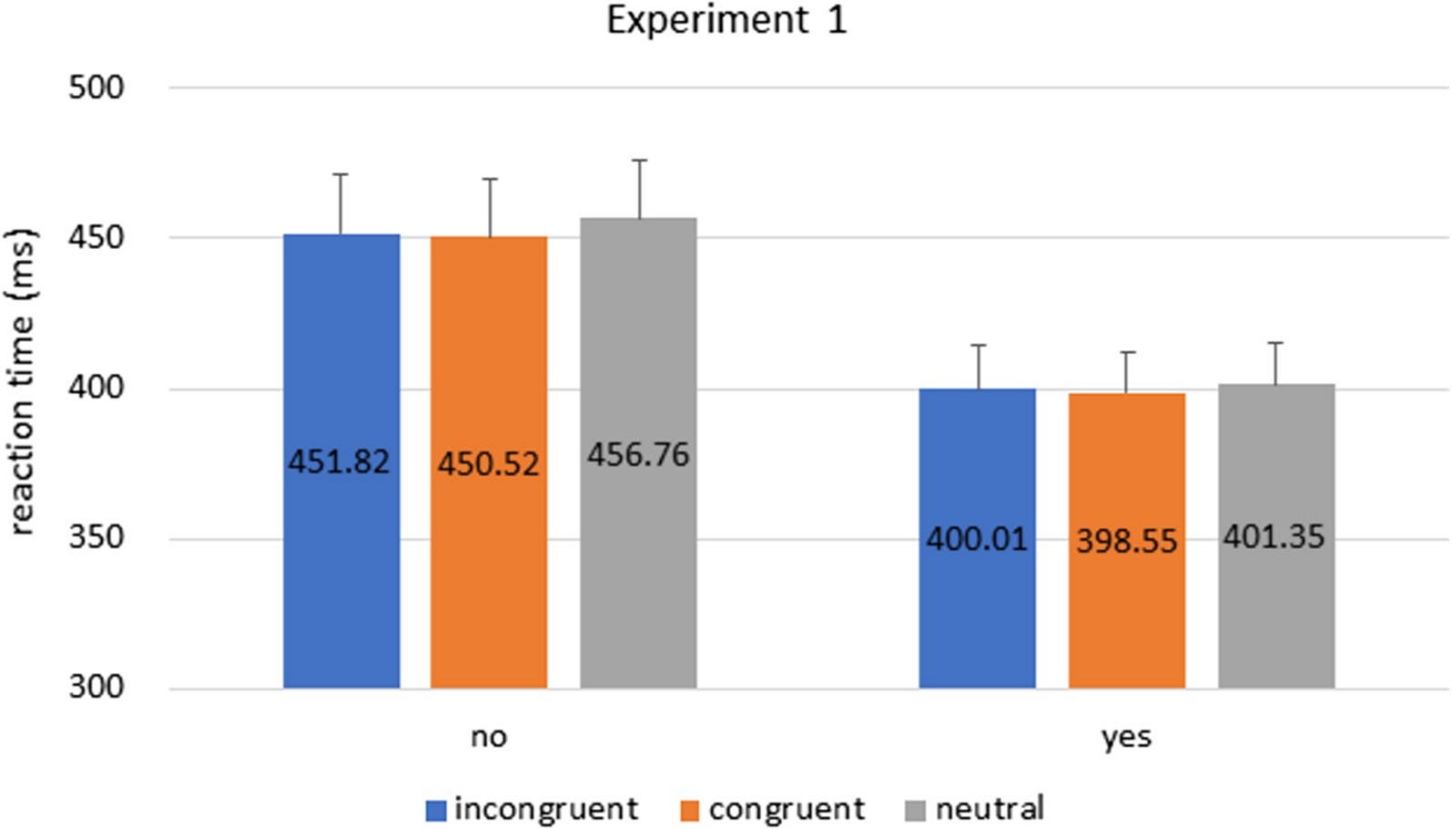
Experiment 1 Results. Bars indicate reaction times in incongruent, congruent, and neutral conditions in ‘yes’ and ‘no’ groups. The overall RT in the ‘no’ group was longer than the ‘yes’ group. Error bars represent standard error of the mean

To answer our research question, participants’ RT were analyzed with a mixed 2 × 3 ANOVA, with cue congruence (congruent vs. incongruent vs. neutral) as within-subject factor, and prior knowledge as between-subject factor (‘yes’ vs. ‘no’ groups). RT from incorrect trials in the cueing task were excluded from analysis. There was a significant main effect of prior knowledge [F(1,125) = 5.276, p = 0.023, η^2^_p_ = 0.040], and nothing else was statistically significant [cue congruence: F(2,250) = 1.389, p = 0.251, η^2^_p_ = 0.011; interaction: F(2,250) = 0.268, p = 0.745, η^2^_p_ = 0.002]. The results showed that RT from the ‘yes’ group was around 50 ms faster than those from the ‘no group’ (Fig. 3). This was true regardless of congruence types.^1^

Our experiment has generated somewhat ambiguous results. That is, we did not observe cue congruence RT facilitation, or incongruent slowing. Instead, there was only a main effect of prior knowledge, where the ‘yes’ group showed faster RT than the ‘no’ group, in congruent condition, incongruent condition, and even neutral condition. This general, non-cue-specific, faster RT seems to reflect some kind of inherent population difference, rather than reflecting our experimental manipulation. Could it be that the prior knowledge question of FedEx arrow is inadvertently tapping into some preexisting population characteristics?

Indeed, it may very well be that people who are well-read, or generally more curious, are the ones more likely to learn more about things like the FedEx arrow. Therefore, the generally-faster RT may simply be an effect of participants’ curiosity or even intelligence, which has been shown to correlate with simple and choice RT (Vernon, 1983). Or perhaps the online experimental platform has introduced much uncontrolled variability to the experiment (e.g., eye-monitor distance, quality of the computer or monitor speed, internet speed…etc.) that otherwise would not occur in the laboratory.

To test these possibilities, we conducted Experiment 2, a replication of Experiment 1 in a controlled laboratory setting. Crucially, knowledge of the FedEx arrow is manipulated—where half of the participants are told about the FedEx arrow beforehand—to see whether the current observations would persist.

## Experiment 2: lab replication

In this lab-based replication, university students were recruited to come into the laboratory, and performed the task in a highly controlled experimental setting. Importantly, because FedEx does not handle local parcels in Taiwan, most of the participants have not heard of the FedEx brand before, and none knew about its logo design. Therefore, in this experiment, half of the participants were told about the FedEx logo design as a fun trivial fact prior to the experiment, and were told to keep it in mind throughout the experiment, though no explicit connection to the experiment was given.

### Methods

#### Participants

Fifty participants (21 male, 29 female, age 20 ~ 34, mean age = 22.29) from Taipei Medical University, who had no prior knowledge of the FedEx logo, took part in the experiment. All had normal or corrected-to-normal eyesight. Half of the participants were randomly assigned to the ‘yes’ group (11 male, 14 female, age 20 ~ 30, mean age = 22.40) and half to the ‘no’ group (10 male, 15 female, age 20 ~ 34, mean age = 22.17). There was no statistically significant age difference between these two groups [t_(48)_ = 0.240, p = 0.811, Cohen’s d = 0.069]. The ‘yes’ group was given a 60 s trivial fact introduction to the FedEx logo design and hidden arrow. Everything else (i.e., stimuli, procedure) was the same between the ‘yes’ and ‘no’ groups. Four participants’ data were excluded (3 from “yes” group and 1 from “no” group) due to their indication of noticing the hidden arrow in the post-experiment survey.

All participants gave written informed consent prior to their participation. All received financial compensation for their time. All experimental procedures were reviewed and approved by the Joint Institutional Review Board of Taipei Medical University, Taiwan.

#### Stimuli and procedure

All stimuli were the same as Experiment 1. Participants sat in a dimly-lit room, and rested their chins on a chinrest 57 cm away from the display. The width and length of the FedEx-like images subtended 8 by 8 degrees of visual angle. The task was written and executed using software E-prime 2.0 (Psychology Software Tools Inc., Pittsburgh, USA). All other procedures were identical to Experiment 1.

Due to the manipulation of prior knowledge, the post-experiment survey was slightly modified from Experiment 1. Participants had to answer three questions: (1) Did you notice anything weird about the images from this experiment? (2) During this experiment, did you notice any white arrow hidden inside the images? (3) Can you point out the location of white hidden arrow in the image on the screen? (this was accompanied by a randomly-selected arrow-present image from the experiment).

### Results and discussion

Our post-experiment questionnaire showed that only 3 out of 25 participants from the ‘yes’ group (88% unaware) and 1 out of 25 from the ‘no’ group (96% unaware) reported noticing the hidden arrow. The remaining 46 reported that they did not notice the hidden arrow throughout the experiment until being asked about it in the post-experiment survey, suggesting a truly implicit nature of negative space. However, when prompted, all were able to correctly locate the arrow.

All participants were attentive to the cues as their overall accuracy of the cueing task and the change detection task was 96.93% and 94.35%, respectively. The respective accuracy from the ‘yes’ group was 97.44% and 93.66%, and from the ‘no’ group it was 96.48% and 94.95%, with no significant differences between them in each task [Cueing**: **t_(45)_ = 0.436, p = 0.665, Cohen’s d = 0.127; Change detection: t_(45)_ =  − 0.915, p = 0.365, Cohen’s d = − 0.267]. Data from incorrect trials in the cueing task were excluded from further analysis.

**Fig. 4:**
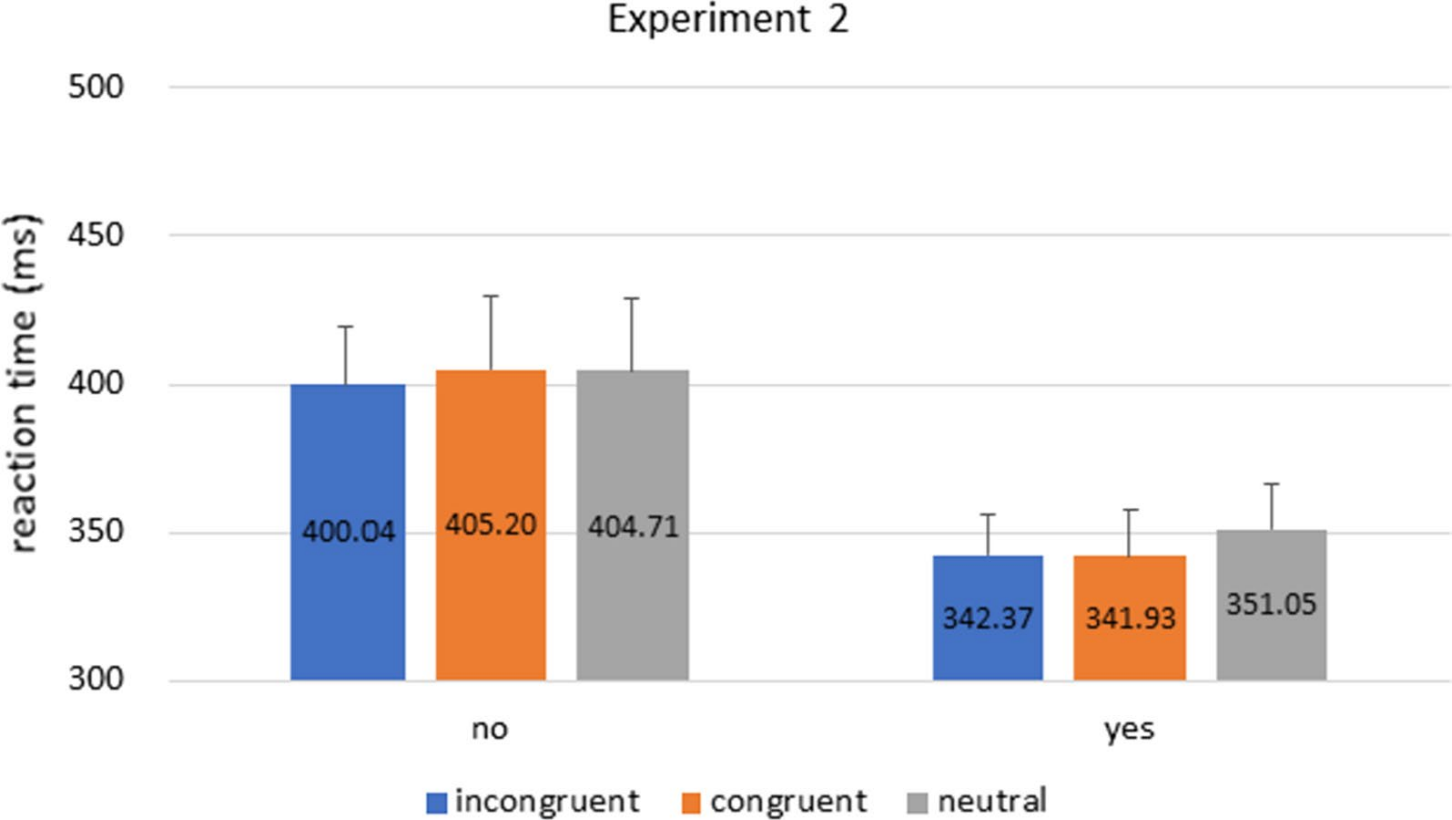
Experiment 2 Results. Bars indicate reaction times in incongruent, congruent, and neutral conditions in ‘yes’ and ‘no’ groups. Like Experiment 1, the overall RT in the ‘no’ group was longer than the ‘yes’ group. Error bars represent standard error of the mean

Participants’ RT were analyzed with a mixed 2 × 3 ANOVA with factors of cue congruence (congruent vs. incongruent vs. neutral) as within-subject factor and prior knowledge as between-subject factor. The results in Experiment 2 replicated Experiment 1 (Fig. 4): there was a significant main effect of prior knowledge [F(1,45) = 4.325, p = 0.043, η^2^_p_ = 0.088], and no main effect of cue congruence or interaction between the factors [cue congruence: F(2,90) = 1.109, p = 0.323, η^2^_p_ = 0.024; interaction: F(2,90) = 0.563, p = 0.531, η^2^_p_ = 0.012].^2^

Just like Experiment 1, the only significant effect here is the faster RT from the ‘yes’ group over the ‘no’ group, with the same magnitude (~ 50 ms), and in congruent, incongruent, and neutral conditions. This lab-based replication is important for two reasons. First, it suggests that our results from Experiment 1 is real, and not due to some kind of participant- or equipment-related variability from the online platform. Second, because we directly manipulated participants’ knowledge of the FedEx arrow here, these results suggest that the faster RT from the ‘yes’ group is not due to some kind of preexisting population difference. Rather, there is a causal relationship between prior knowledge of the hidden arrow (either self-acquired or taught by the experimenter immediately before the experiment) and the subsequent faster RT.

The faster, yet nonselective (in terms of congruence) RT, seems to suggest that participants from the ‘yes’ group were able to process *all* FedEx-like cueing images faster, or with more ease. From the figure-ground segregation literature, it is known that multiple shapes would initially compete for figural status, and the subsequent winner becomes the figure while the loser becomes shapeless ground. The Biased Competition Model further posits that the background, after losing competition, is suppressed to facilitate processing for the figure (e.g., Peterson & Skow, 2008). As such, it is possible that the ‘yes’ participants could somehow resolve this figure ground competition faster, perhaps not by looking at the arrows, but by ignoring or suppressing them (as a part of the background). Doing so would give the participants more time or more attentional resources to process the first cueing image (though not the arrow), which, from the participants’ perspective, is their primary (and perhaps also more challenging) task.

Another similar explanation is that, to facilitate change detection performance, perhaps people who knew about the negative space could segregate (but not suppress) figure and ground, or orange and white pixels, in a binary manner. In this case, all images can be processed and remembered as orange vs. white shapes (memory load = 2), instead of 3 alphanumeric symbols (memory load = 3). This would effectively reduce “yes” participants’ memory load, and would also predict a nonselective speeding in RT as the white arrow is now a part of the overall background. Indeed, research has shown that global processing is preferred for images up to approximately 7 degrees of visual angle (Kinchla & Wolfe, 1979), which is very close to the present setup of 8 by 8 degrees.

The present experiment is unable to differentiate these two “how” accounts on the faster RT performance from our ‘yes’ participants. But regardless of “how”, it seems that the answer to our original “what” question—do people really unconsciously perceive the hidden FedEx arrow?—is a firm “no”. That is, without a selective speeding RT effect in the congruent condition, it is likely that the meaning of the arrow was never picked up by the visual system.

To substantiate this conclusion, we have to make sure that the congruence effect would indeed emerge when it is picked up by the visual system in our task design. To confirm this, in Experiment 3 we colored the FedEx arrow blue so that it becomes somewhat obvious by not blending into the foreground or background.

## Experiment 3: blue arrow

**Fig. 5:**
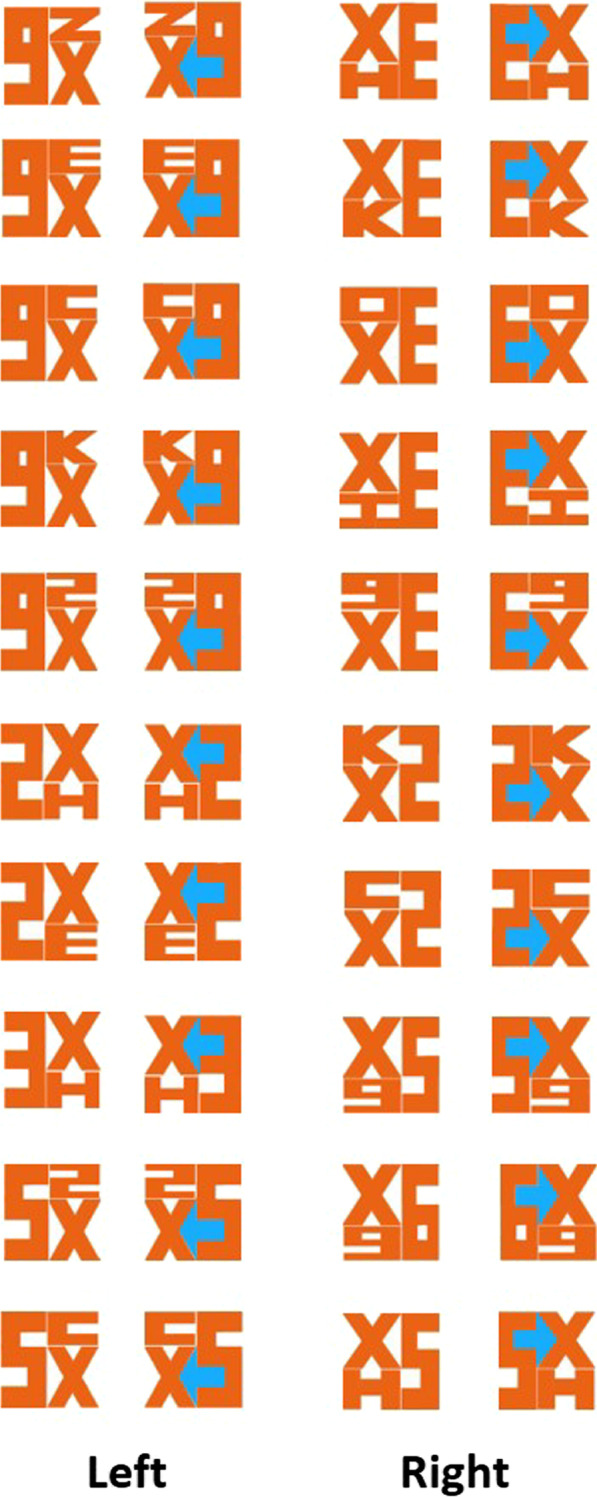
Partial stimuli from Experiment 3. All left, right, neutral stimuli (not shown), as well as their reverse controls, were the same as in Experiment 1 and 2, except that the arrows, when present, were highlighted in blue to contrast with the orange alphanumeric characters

In this Experiment, the FedEx arrows were painted blue so that it is no longer “invisible” as a part of the background (Fig. 5). Although this explicit emphasis on the arrow deviates from the original figure-ground question (and perhaps into the attentional selection and filtering literature), the current experiment nonetheless serves as an important control experiment to ensure that the task and stimuli can induce a congruence cueing effect when the arrow is explicitly picked up by the visual system. In addition, either from a figure-ground biased competition perspective, or from an attentional selection perspective, this highlight should give the arrow some competitive advantage for visual processing.

### Methods

#### Participants

Eighty-one participants (52 male, 28 female, age 28 ~ 69, mean age = 45.27) participated via Amazon Mechanical Turk. Seventeen were excluded due to < 70% accuracy in either the cueing task (n = 12), change detection task (n = 2), or both (n = 3). Of the remaining 64 individuals, post-experiment survey showed that 41 indicated knowing about the FedEx arrow beforehand (“yes” group: 26 male, 15 female, age 28 ~ 62, mean age = 44.07), and 23 did not (“no” group: 15 male, 8 female, age 34 ~ 69, mean age = 46.22). There was no age difference between the two groups [t_(62)_ = − 0.826, p = 0.412, Cohen’s d = − 0.215]. A total of 51 participants (79.69%) reported seeing the blue arrow (32 from ‘yes’ group, 19 from ‘no’ group), but were not eliminated from further analysis as we reasoned the purpose of making the arrow blue was to make the arrow explicit to the visual system. As such, manipulating cue explicitness while eliminating participants who could pick up explicit cues seems unsuitable in the context of the present experiment and the current set of images (see footnote 3 for the same analysis for Experiment 1 and 2), and would leave us with insufficient number of participants.

All participants gave informed consent via mouse click prior to their participation, and all received financial compensation for their time. All experimental procedures were reviewed and approved by the Joint Institutional Review Board of Taipei Medical University, Taiwan.

#### Stimuli and procedure

Same FedEx-like figures from Experiment 1 and 2 were used here. But, whenever the arrows were present, they appeared in blue to create a clear contrast with the orange alphanumeric stimuli (Fig. 5). All procedures were identical as Experiment 1 and 2.

The post-experiment survey is the same as Experiment 1, except that we added one forced-choice question regarding participants’ strategy for remembering the FedEx-like figures. Participants were asked whether they 1) memorized the three alphanumeric characters individually, or 2) memorized the entire figure holistically.

### Results and discussion

All participants were attentive to the cues as their overall accuracy for the cueing task and change detection was 98.58% and 93.17%, respectively. The respective accuracy in the ‘yes’ group was 98.87% and 93.85%, and the ‘no’ group was 98.07% and 91.96%, with no significant differences between them in each task [Cueing**: **t_(62)_ = 1.556, p = 0.125, Cohen’s d = 0.405; Change detection: t_(62)_ = 1.503, p = 0.138, Cohen’s d = 0.392]. Data from incorrect cueing trials were excluded from subsequent analysis.

Post-experiment survey asking for participants’ memory strategy showed that, in the ‘yes’ group, 27 participants (65.85%) reported memorizing the alphanumeric items individually and 14 participants (34.15%) reported memorizing the entire figure holistically. In the ‘no’ group, the numbers were 12 (52.17%) and 11 (47.83%), respectively. However, these two ratios did not differ significantly [X^2^(1, N = 64) = 1.158, p = 0.282].

Participants’ RT were analyzed with a mixed 2 × 3 ANOVA with factors of cue congruence (congruent vs. incongruent vs. neutral) as within-subject factor and prior knowledge as between-subject factor.^3^ Results showed that there was a significant main effect of prior knowledge [F(1,62) = 7.345, p = 0.009, η^2^_p_ = 0.106], and no main effect of cue congruence or interaction between the factors [cue congruence: F(2,124) = 0.842, p = 0.143, η^2^_p_ = 0.013; interaction: F(2,124) = 0.699, p = 0.499, η^2^_p_ = 0.011].^4^

**Fig. 6:**
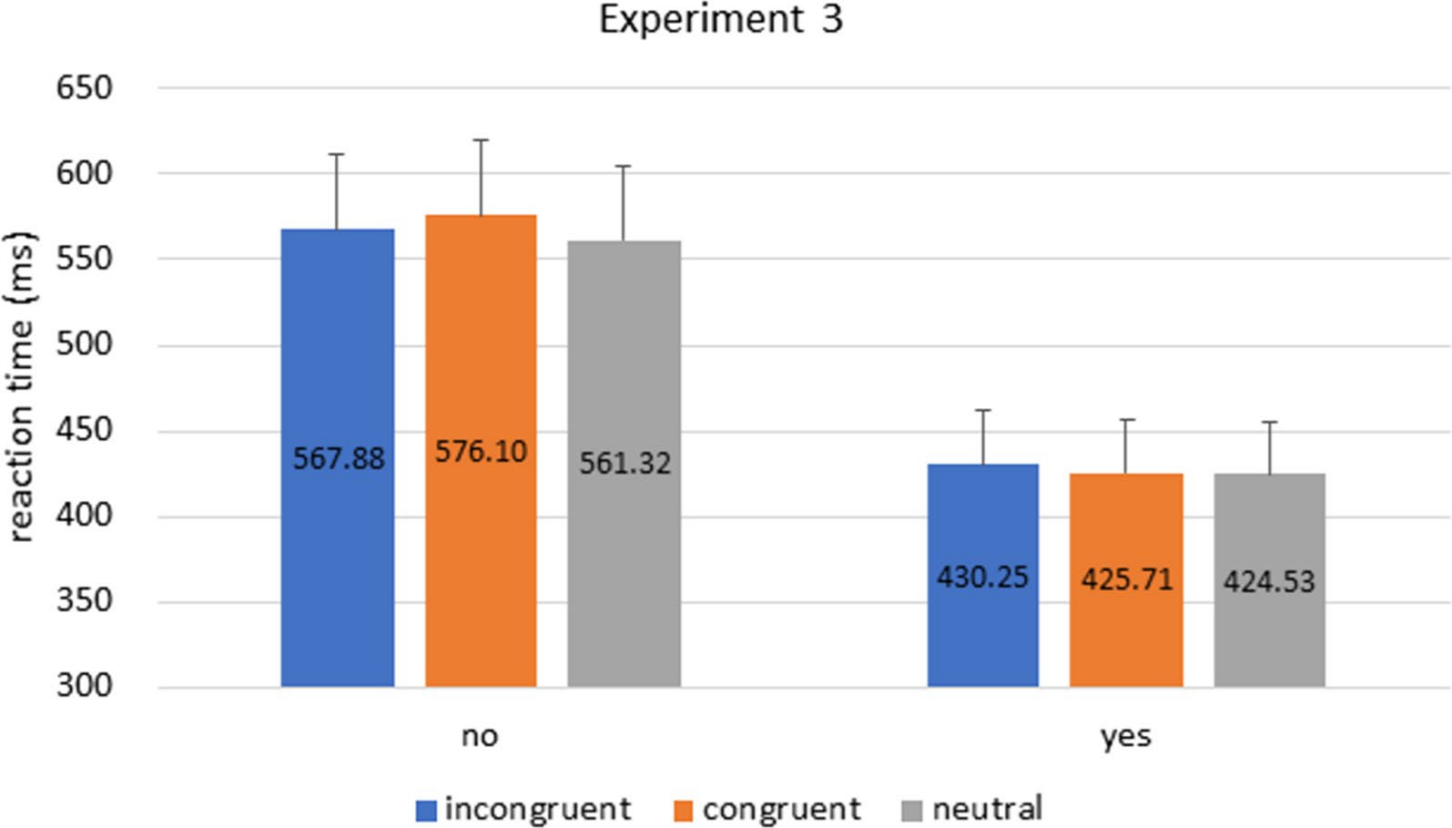
Experiment 3 Results. Bars indicate reaction times in incongruent, congruent, and neutral conditions in ‘yes’ and ‘no’ groups. Like Experiment 1 and 2, the ‘yes’ group had faster RT than the ‘no’ group across all congruence conditions. Error bars represent standard error of the mean

In contrast to our prediction (i.e., emergence of the congruent cueing effect), here we again replicated results from Experiment 1 and 2 (Fig. 6). In other words, our ‘yes’ participants who knew about the FedEx logo beforehand was again faster in all congruence conditions without selectivity for the congruent direction, despite the fact that all FedEx arrows have been highlighted blue. This replication suggests that the lack of congruence effect from Experiment 1, 2, and here, may have been driven by something else in our task design, and does not fully reflect a lack of unconscious arrow processing.

One possibility, and also a common factor across all 3 experiments so far, is our dual task design. That is, although the change detection task was implemented to encourage participants to pay close attention to the FedEx-like figures, from the participants’ perspective it has become their primary task as it was a more effortful task compared to left/right cueing. As such, to ensure accurate memory of the (orange) figure, the change detection task demand may have motivated participants to suppress all unnecessary information, whether white or blue, as uninformative or irrelevant background. This task demand idea is still consistent with the nonselective background-suppression account (that only exists in those who knew about the FedEx arrow) we proposed in earlier experiments, but adds a “why” to the explanation (i.e., task demand from change detection).

If our speculation above about task demand is true, this would suggest that when the change detection task is removed, the background-suppression effect from the ‘yes’ group should also be eliminated (or substantially weakened). Furthermore, the congruence effect should also emerge if the arrow can now be picked up by the visual system. This is the purpose of Experiment 4a (white arrow without change detection) and 4b (blue arrow without change detection).

As a side note, data from this experiment perhaps also adds credence to the background-suppression account slightly over the perceptual load account mentioned in previous discussion. This is because the current setup, by highlighting the arrow blue, now consists of 3 colors in each arrow-present image. Therefore, the perceptual load account would predict faster RT in neutral (i.e., 2 colors) trials over congruent or incongruent (i.e., 3 colors) arrow-present trials in the ‘yes’ group, though it is not currently observed.

## Experiment 4a: invisible arrow without change detection

In Experiment 1~3, participants’ pressure to memorize the first FedEx-like figure may have encouraged the ‘yes’ group to perform figure-ground segregation faster and suppress the background more aggressively (to enhance memory for the figure). Thus, the change detection task that we have originally implemented to facilitate the cueing effect may have backfired and eliminated it instead. To this end, here we conducted Experiment 1 again, but with only the cueing task and without change detection. An elimination or substantial weakening of the faster RT from the ‘yes’ group would confirm our suspicion of task demand, and provide further evidence for the background-suppression account. Independently, a faster RT in the congruent condition, either in the ‘yes’ group or in all participants, would confirm unconscious processing of the FedEx arrows.

### Methods

#### Participants

Two hundred and forty-one participants (142 male, 99 female, age 20~68, mean age = 42.66) from the United States participated via Amazon Mechanical Turk. The experiment was introduced as a speeded test of attention, and nothing about the FedEx arrow was mentioned in the description. Twenty-one participants’ data were excluded from analysis due to < 70% accuracy in the cueing task. Additional 11 participants (10 from ‘yes’ and 1 from ‘no’ group) were excluded because they noticed the arrows in the FedEx-like figure while performing the experiment.

In the remaining 209 participants, based on their post-experiment survey, 92 participants indicated knowing about the FedEx arrow beforehand (‘yes’ group: 60 male, 32 female, age 20 ~ 68, mean age = 41.27) and 117 did not (‘no’ group: 66 male, 51 female, age 21 ~ 68, mean age = 44.07). There was no significant difference in age between the two groups [t_(207)_ =  − 2.028, p = 0.044, Cohen’s d = − 0.283].

All participants gave informed consent via mouse click prior to their participation, and all received financial compensation for their time. All experimental procedures were reviewed and approved by the Joint Institutional Review Board of Taipei Medical University, Taiwan.

#### Stimuli and procedure

**Fig. 7:**
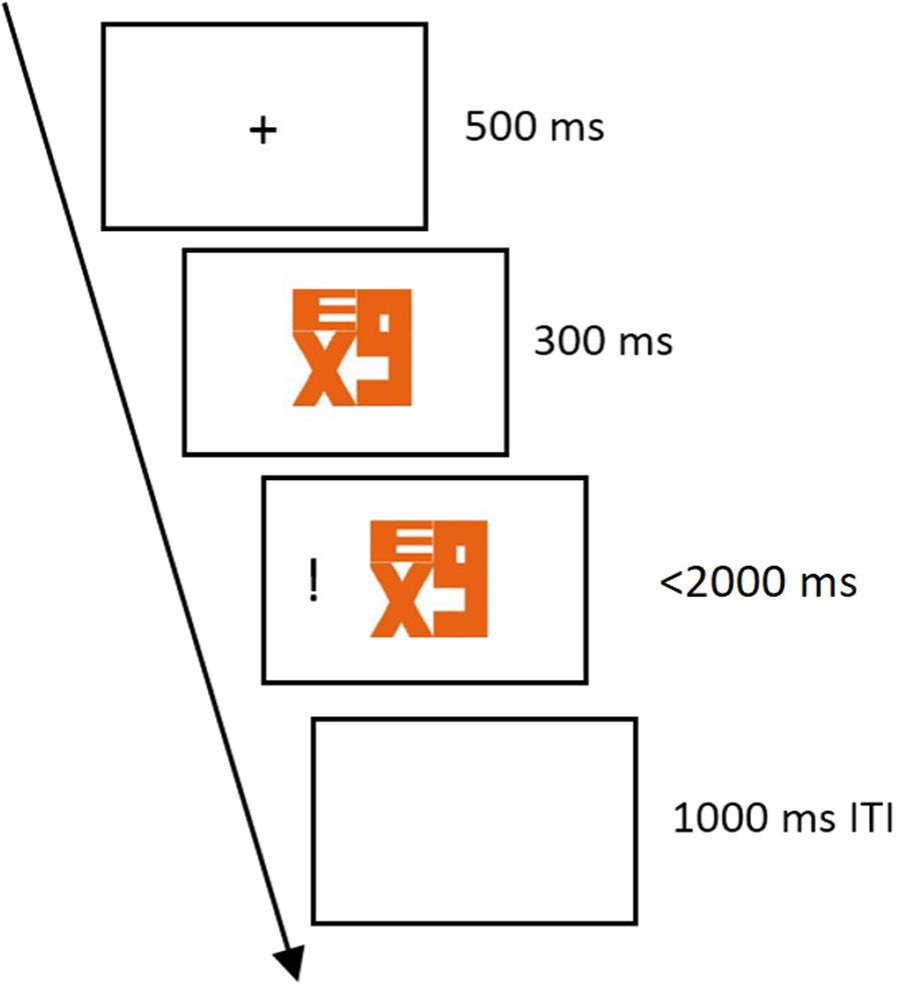
Experiment 4a Procedure. Participants were to press the button according to the location of the target (i.e., exclamation mark), which can be congruent or incongruent with the cued direction

Same FedEx-like figures from previous experiments were used here. The trial started with a 500 ms fixation cross, followed by a 2300 ms FedEx-like figure. The target exclamation mark would appear either at the left or right side, at 300 ms after onset of the FedEx-like figure. Participants had up to 2000 ms to respond, and the trial would end and go into 1000 ms ITI as soon as a response is recorded (Fig. 7).

### Results and discussion

All participants performed the task well and at similar performance levels to what is observed in Experiments 1–3. Their overall accuracy for the cueing task was 97.99%. Accuracy was 97.77% in the ‘yes’ group, and 98.16% in the ‘no’ group, and there was no significant difference between the two groups [t_(207)_ =  − 1.142, p = 0.255, Cohen’s d = − 0.159]. Mean accuracy in each cue congruence conditions for the ‘no’ group was 98.28%, 98.40%, and 98.09% in incongruent, congruent, and neutral conditions, respectively. For the ‘yes’ group, respective mean accuracy was 97.68%, 97.90%, and 97.70%. Data from incorrect trials were excluded from RT analysis.

**Fig. 8:**
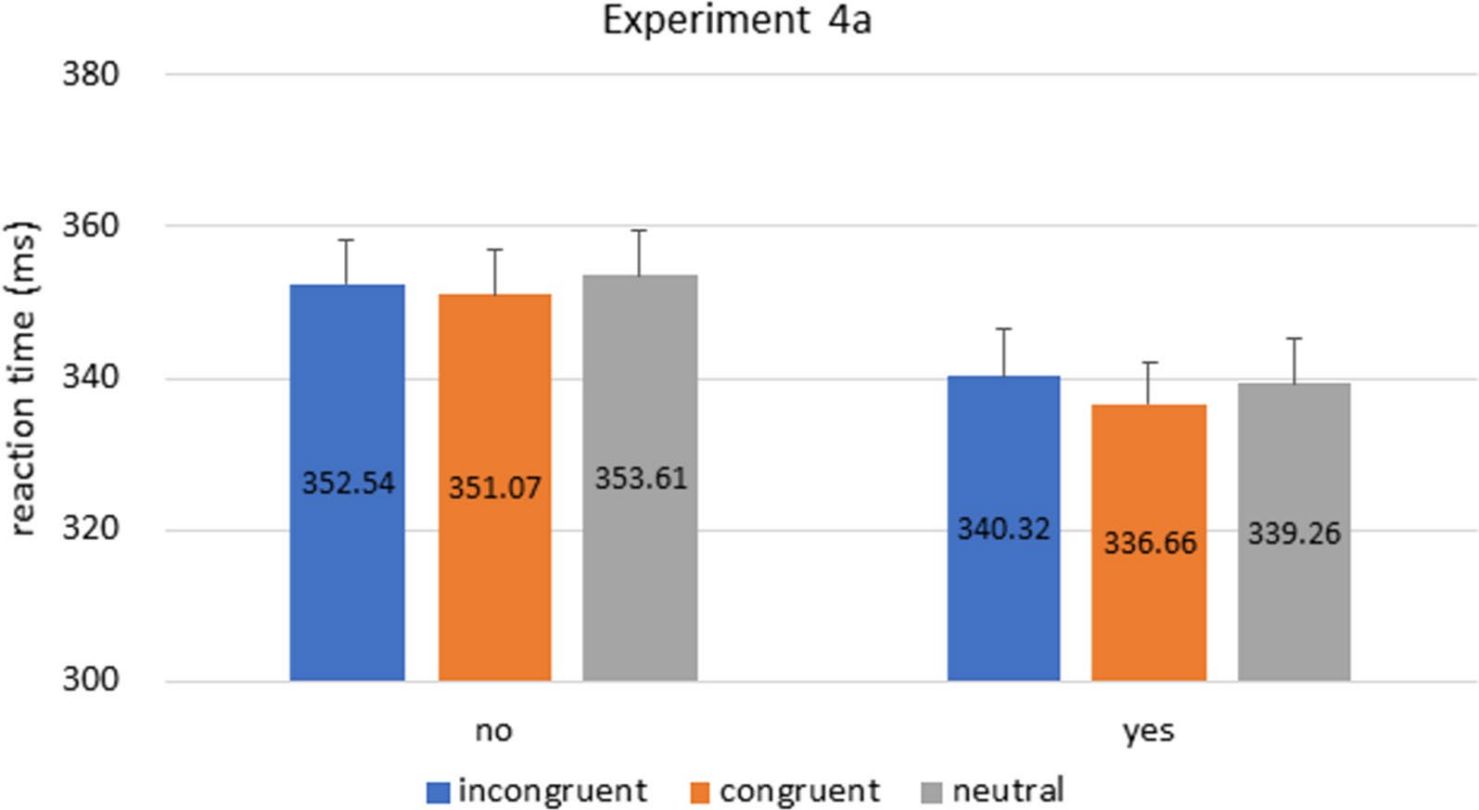
Experiment 4a Results. Bars indicate reaction times in incongruent, congruent, and neutral conditions in ‘yes’ and ‘no’ groups. Similar to previous experiments, the overall RT in the ‘yes’ group was faster than the ‘no’ group, but to a lesser extent (~ 10 ms) and only marginally significant. Error bars represent standard error of the mean

Participants’ RT were analyzed with a mixed 2 × 3 ANOVA, with cue congruence (congruent vs. incongruent vs. neutral) as within-subject factor, and prior knowledge as between-subject factor (‘yes’ vs. ‘no’ groups). Unlike previous experiments, the main effect of prior knowledge is now only marginally significant [prior knowledge**: **F(1,206) = 3.633, p = 0.058, η^2^_p_ = 0.017], and cue congruence was not significant; cue congruence: F(2,412) = 0.757, p = 0.451, η^2^_p_ = 0.004]. Additionally, the interaction between cue congruence and prior knowledge was not statistically significant [F(2,412) = 0.147, p = 0.831, η^2^_p_ < 0.001] (Fig. 8).^5^

The present single task design is perhaps a more suitable test for the implicit processing of the FedEx arrow. Now, without task demand to suppress the background and remember the figure, our ‘yes’ group showed only a marginally faster RT than the ‘no’ group. It is also important to note that this mildly faster RT is (1) only achieved using twice as much participants than Experiment 1, (2) with a much smaller effect size (~ 10 ms compared to ~ 50 ms in previous experiments), and (3) only marginally significant (p = 0.058).

Together, we take this as evidence suggesting that the nonselectively faster RT from the ‘yes’ group from Experiment 1~3 was mainly driven by our dual task design, where the change detection task demand encouraged ‘yes’ participants to suppress the irrelevant background more actively. This finding is still interesting, as it highlights that knowing something about the FedEx arrow, or negative space in general, allowed the ‘yes’ participants to do figure-ground segregation faster despite that almost all of them did not report seeing the arrow during the experiment (~ 4% in Experiment 1, and ~ 10% here).

The weakening of the faster RT in the ‘yes’ group suggests that task demand is no longer an issue, yet we still did not observe a congruence effect. There seems to be a 3 ms advantage in the congruent condition from the ‘yes’ group, but not statistically significant. Therefore, results from the current experiment seems to suggest that the answer to our original question is still a “no”.

## Experiment 4b: blue arrow without change detection

**Fig. 9:**
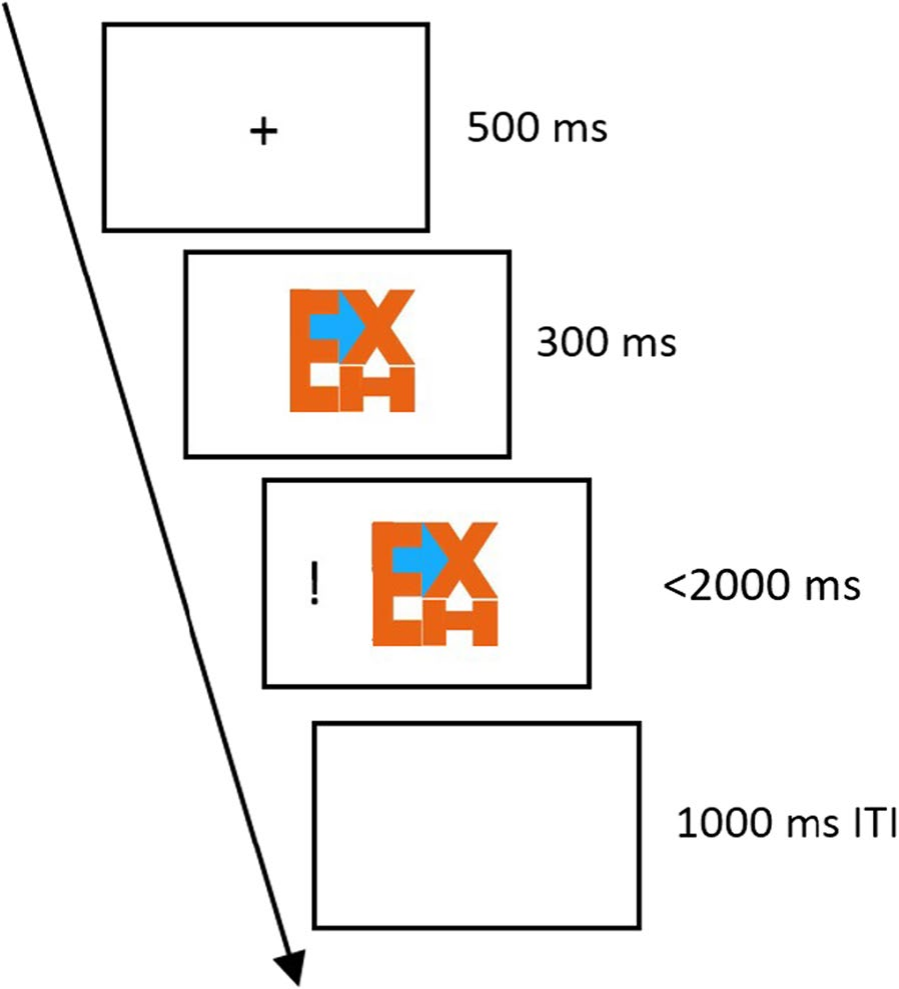
Experiment 4b Procedure. Participants were to press the button according to the location of the target (i.e., exclamation mark), which can be congruent or incongruent with the cued direction. The arrows were highlighted in blue to contrast with the orange alphanumeric items

In this experiment we aim to retest what we set out to do in Experiment 3—to give the arrow a more competitive edge by highlighting it blue—but this time with a cleaner single task design (Fig. 9). With participants no longer under pressure to resolve figure-ground competition rapidly, and that the arrow is highlighted in blue, we expect a congruency cueing effect either in the ‘yes’ group, or in both ‘yes’ and ‘no’ groups. A congruency effect here would also confirm that our lack of cueing effect in Experiment 4a was a valid no-effect.

### Participants

One hundred and sixteen participants (73 male, 43 female, age 23 ~ 79, mean age = 43.71) participated via Amazon Mechanical Turk. Six participants’ data were excluded from analysis due to < 70% accuracy in the cueing task, and two participants indicated that they did not understand/follow the instructions in the exit survey.

Of the remaining 108 participants, 57 indicated knowing about the FedEx arrow beforehand (“yes” group: 38 male, 19 female, age 23 ~ 69, mean age = 44.23) and 51 did not (“no” group: 29 male, 22 female, age 32 ~ 65, mean age = 43.14). There was no significant difference in age between the two groups [t_(106)_ = 0.581, p 0.563, Cohen’s d = 0.112]. A total of forty-six (42.59% of all participants) indicated noticing the blue arrow during the experiment (31 in ‘yes’ group, 15 in ‘no’ group). Like Experiment 3, because the arrow is no longer a part of the background due to explicit manipulation, and that almost 50% of participants noticed the arrow, these participants were not excluded from subsequent analysis (see footnote 6 for the same analysis for Experiment 4a).

All participants gave informed consent via mouse click prior to their participation, and all received financial compensation for their time. All experimental procedures were reviewed and approved by the Joint Institutional Review Board of Taipei Medical University, Taiwan.

### Stimuli and procedure

All stimuli and procedures were identical to Experiment 4a, except that the FedEx arrows were highlighted in blue to contrast with the orange alphanumeric items.

### Results and discussion

All participants performed the task well and at similar performance levels to what is observed in Experiments 1–3. Their overall accuracy for the cueing task was 97.38%. Accuracy was 96.95% in the ‘yes’ group, and 97.87% in the ‘no’ group, and there was no significant difference between them [t_(106)_ =  − 1.120, p = 0.265, Cohen’s d = − 0.216]. Mean accuracy for the ‘no’ group was 97.51%, 97.75%, and 98.12% in incongruent, congruent, and neutral conditions, respectively. For the ‘yes’ group, respective mean accuracy was 95.98%, 97.24%, and 97.11%. Data from incorrect cueing trials were excluded from subsequent RT analysis.

**Fig. 10:**
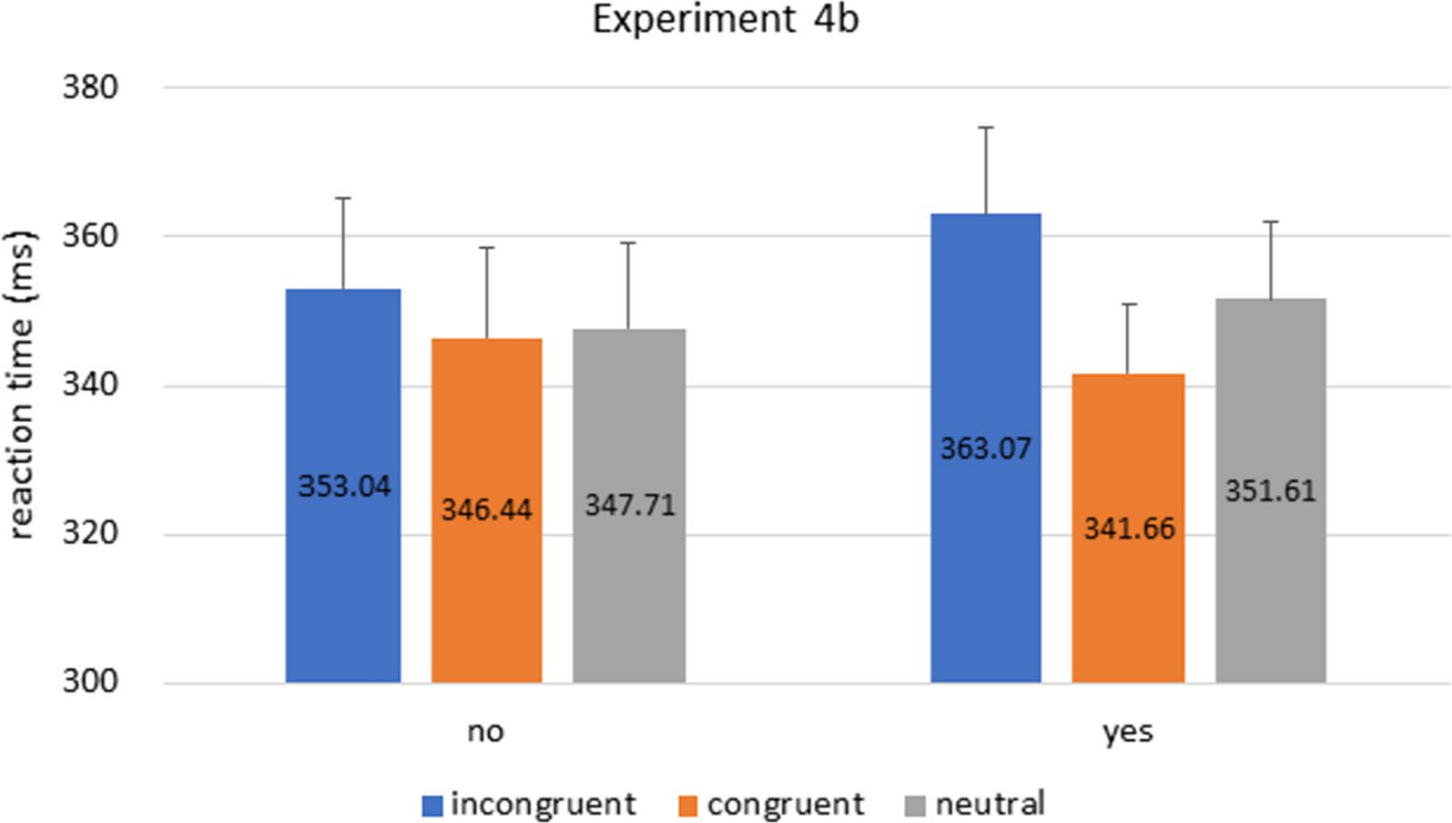
Experiment 4b Results. Bars indicate reaction times in incongruent, congruent, and neutral conditions in ‘yes’ and ‘no’ groups. The RT for congruent trials were significantly faster than RT for incongruent trials. No significant RT difference between ‘yes’ and ‘no’ groups was observed. Error bars represent standard error of the mean

Participants’ RT were analyzed with a mixed 2 × 3 ANOVA, with cue congruence (congruent vs. incongruent vs. neutral) as within-subject factor, and prior knowledge as between-subject factor (‘yes’ vs. ‘no’ groups).^6^ The main effect of cue congruence was significant [cue congruence**: **F(2,212) = 6.185, p = 0.006, η^2^_p_ = 0.055], and the main effect of prior knowledge was not [F(1,106) = 0.041, p = 0.840, η^2^_p_ < 0.001], or the interaction between cue congruence and prior knowledge [F(2,212) = 1.724, p = 0.189, η^2^_p_ = 0.016]^7^ (Fig. 10).

To further explore the observed congruence effect, post-hoc analysis with Holm correction showed that RT for incongruent trials was longer than RT for congruent trials [t_(107)_ = 3.494, p = 0.002]. There was no statistical differences between neutral and the other two conditions [incongruent vs neutral**: **t_(107)_ = 2.095, p = 0.075; congruent vs neutral: t_(107)_ = − 1.399, p = 0.163]. This is congruent-incongruent difference is consistent with previous reports that utilized nonpredictive arrow cues in the context of a cueing paradigm (Hommel et al., 2001).

The key observation from this experiment is that our ‘yes’ participants are no longer faster than their ‘no’ counterparts. We take this as evidence suggesting that, although the ‘yes’ participants could do figure-ground segregation much faster due to their prior knowledge of the negative space (as demonstrated in Experiment 1~3), such perceptual processing was no longer put in action when (1) the task demand for an “orange figure” is eliminated (difference between this experiment and Experiment 1~3), and (2) the arrow is no longer hidden as a part of the white background (difference between this experiment and Experiment 4a). And, it seems that these two conditions need to both be present, in order for the congruence cueing effect to emerge. Therefore, contrasting with Experiment 4a, it seems that our participants simply could not utilize the white arrows as cues.

## General discussion

In this study we used FedEx-like images with hidden arrows to serve as endogenous arrow cues in a Posner’s cueing task. The prediction is straight-forward: a cueing effect would suggest subliminal processing of the hidden arrow, and vice versa. In short, we did not observe any cue congruency effect. However, there was an overall effect of prior knowledge: when people knew about the possibility of negative space and hidden arrow, their RTs were generally faster in neutral, congruent, and even incongruent conditions, suggesting a general background suppression as opposed to cue processing. This was true in participants from North America who had heard of the FedEx arrow before (Experiment 1), and also in our Taiwanese sample who were just informed of such design technique immediately prior to the experiment (Experiment 2). Strangely, such speeding effect persisted even when we turned the arrow blue (i.e., explicit; Experiment 3), which implied that the effect was likely driven by the task demand of our primary change detection task. Indeed, when we eliminated the change detection task from our design so that the task demand for background suppression is low, the speeding RT effect in the ‘yes’ group weakened and became non-significant when the arrow was implicit (Experiment 4a), and was entirely eliminated and replaced by a significant congruent cueing effect when the arrow was blue and explicit (Experiment 4b). Interestingly, despite prior knowledge of the FedEx arrow, most participants from the ‘yes’ group did not realize the presence of the hidden arrow in our figures, suggesting the implicit nature of the speeded RT effect. Together, these results suggest that people who are informed about the possibility of negative space can implicitly suppress such negative space (i.e., background) more effectively (Experiment 1, 2, 3), but cannot process the semantics of the background (i.e., arrow) unless it is made explicit (Experiment 4).

### Implicit cueing

Many studies have already proven arrows to be powerful endogenous cues when used explicitly. For example, Hommel and colleagues (2001) used non-predictive cues that were displayed at the center, and found faster RT when arrow direction and target location were congruent. This RT facilitation effect was still present even when participants are explicitly told by the experimenter that the arrows are non-predictive (Tipples, 2002). What sets the current study apart, then, is that the arrow is also presented centrally, but as part of the background. Previous studies have already suggested that figure-ground segregation can be achieved implicitly and automatically (Kimchi & Peterson, 2008). Furthermore, implicitly processed arrows (via masking) can be processed to an extent that can induce priming and inverse priming effects (Verleger et al., 2004). However, most studies have focused on the perceptual fate of the figure, where unconsciously processed (e.g., backward masking, continuous flash suppression) or unattended (e.g., inattentional and change blindness) figures are able to prime certain behavioral responses. Yet, in the case of the FedEx logo, the arrow is actually part of the background, whose shape happens to carry symbolic directional information. Therefore, in this study we are essentially asking whether people process, or extract meaning from, a background that they are not aware of. It is important to note that the FedEx logo is ideal in this scenario because the arrow literally is the background space, and it contains no features except a blank space that is the same color as the general background (as opposed to other experimental paradigms where background is loosely defined as objects presented in the periphery or away from the center of focused attention).

The closest study to our experiment design that we can find is a conference abstract presented at the Vision Sciences Society (Todd & Marois, 2007). In this study, Todd and Marois displayed a backward-masked arrow, then an explicit arrow cue, in the context of Posner’s cuing task to test whether subliminal arrows can influence endogenous attention. They observed a prime-cue congruency effect only when participants were aware of the prime. Similarly, another study found that when cue predictiveness is being manipulated (predictive: 80% valid; non-predictive: 50% valid), only those participants who are aware of the cue informativeness could take advantage of the cue and exhibit endogenous effect (Bartolomeo et al., 2007). Although we did not use subliminal presentation of the arrow (Todd & Marois, 2007), nor did we manipulate cue predictiveness (Bartolomeo et al., 2007), our results are somewhat consistent with these studies. That is, only participants who are *informed* (but not quite *aware*) of the possibility of negative space could show faster RT. Otherwise, the arrow has to be made completely explicit (Experiment 4b). One important difference, though, is that our observed faster RT from Experiment 1~3 is not exclusive to the congruent condition. Rather, participants from the ‘yes’ group showed faster RT in congruent, incongruent, and also neutral conditions. This lack of directionally-specific cueing effect, in the form of congruent RT facilitation or incongruent RT impairment, seems to suggest that our participants are not automatically perceiving the hidden arrow, at least not enough to facilitate or hinder endogenous attentional orienting. This is unfortunate but perhaps not too surprising. One study by Brintazzoli et al. (2012) primed participants, either consciously or unconsciously, with brand logos (e.g., McDonald’s logo) and observed faster RT to semantically related words (e.g., hamburger), but only in the conscious priming condition and not in the unconscious condition. The authors concluded that perhaps “brand logos could only influence behavior when they were presented clearly above the consciousness threshold” (Brintazzoli et al., 2012).

### Ground suppression

But, if unconscious processing of the hidden arrow is not possible, why should our participants who knew about the arrow (i.e., ‘yes’ group) show faster RT, regardless of arrow direction and congruency? One plausible explanation is that people who knew about the possibility of negative space can achieve figure-ground segregation faster, not by looking at the arrows, but by ignoring them (as a part of the background). Although this is not our initial hypothesis, these results are indeed well predicted by the Biased Competition Model in figure-ground research, which posits that multiple shapes would compete for figural status, and the winner becomes the figure while the loser becomes shapeless ground (e.g., Peterson, de Gelder, Rapcsak, Gerhardstein, & Bachoud-Lévi, 2000). Studies have suggested that such competition takes place in early visual cortices such as V1, V2, and V4 in the 100–160 ms (Neri & Levi, 2007), 250–280 ms (Heinen et al., 2005) time windows, and the strength of such suppression can increase for highly competitive groundside shapes (Cacciamani et al., 2015, 2023). To apply this model to the present study, our ‘yes’ group participants were probably suppressing all white spaces as backgrounds (Experiment 1, 2), or all non-orange spaces as backgrounds (Experiment 3), which would successfully reduce their processing load (i.e., less pixels to process). This suppression account would not only explain the observed faster RT, but would also explain why such faster RT can occur nonselectively in both congruent and incongruent, or even neutral, conditions.

Additionally, the nonselective speeding from Experiment 1–3 seems to suggest a general (and possibly preattentive) mechanism that blocks out the arrow background early, leaving behind no trace of its cueing effect. This implies that the competition in our study is perhaps heavily biased towards the orange figure. This hasn’t always been the case in the literature. For example, Peterson and Skow (2008) showed their participants a 50-ms silhouette that depicted an object category (e.g., house, animal) either with its background (Exp 1) or figure (Exp 2), and found that subsequent familiar/novel object decisions were slower for categories that were “primed” by groundside, and vice versa, suggesting a rapid suppression of the background and its related concepts. However, such concept-specific suppression of the ground suggests that the ground information is still processed before suppression occurs. Similarly, Wager et al. (2015) examined ground suppression and its interaction with attention by cleverly displaying the Eriksen flanker task over figure-ground stimuli. In that study, the central target arrow is always displayed inside a figure, and the flanking distractors inside the background region. These authors observed that incongruent flankers influenced target identification less due to ground suppression, but such suppression was absent for facilitative congruent flankers, suggesting a highly interactive and attentionally selective figure-ground competition process (also see Vecera & O’Reilly, 1998). This is somewhat different from what we found in the present study, as our ‘yes’ group did not show a reversed cueing effect (or any cueing effect) from the arrows whenever there is a dual task design. In light of results from Experiment 4, we now know that perhaps figure-ground segregation is resolved too quickly in our ‘yes’ participants from Experiment 1–3 due to heavily biased competition such as task demand. That is, because our participants’ primary task was to remember the image for change detection, the ‘yes’ group participants who were aware of the possibility of negative space probably engaged in earlier suppression of the potentially-distracting background. Indeed, previous studies have already demonstrated that figure-ground competition can be biased by past experience (Peterson & Gibson, 1994), priming (Hulleman et al., 2005), exogenous attention (Vecera et al., 2004), and most importantly, task set (Peterson, 2015), and our results from Experiment 1–3 echo these points.

To test the idea of task demand being responsible for the fast suppression in Experiment 1 and 2, in Experiment 3 we turned the arrow blue to give the arrow some competitive edge. This manipulation, however, did not change anything and we observed the same fast RT in the ‘yes’ group, regardless of cue congruence. In Experiment 4a, we provided a stronger test by completely eliminating the change detection task, thereby eliminating the task demand to suppress. Indeed, results from Experiment 4a showed the same speeding RT in the ‘yes’ group, but such trend was no longer significant. However, there was still no cueing effect. Cueing effect finally emerged when (1) change detection task demand to suppress was no longer present, and (2) the arrow was no longer the part of the background (i.e., blue) (Experiment 4b). Together, these results suggest that the strength of background suppression can be a function of task demand. This seems to reconcile the different findings between our study and previous studies that have found some effect from the suppressed ground, such as Wager et al. (2015). Particularly, Wager et al. (2015) did not use a dual-task design, whereas we had a demanding “primary” task (i.e., to our subjects, the change detection was the primary task, and the cueing task was secondary).

It is important to note that, even when change detection task was removed, our participants still did not show a cueing effect (Effect 4a). In fact, the RT trend resembled those from Experiment 1–3, only nonsignificant. Perhaps the important finding is from Experiment 4b, where cueing effect is finally observed when the arrow is made explicit. Therefore, to answer our original question of whether people do see the invisible FedEx arrow, the answer (at least in the context of a Posner’s paradigm) seems to be a ‘no’.

### Conclusion

To conclude, in this study we set out to answer “do people really unconsciously perceive the hidden FedEx arrow?” by using FedEx-like images with hidden arrows as an implicit endogenous cue. We found no cueing effect (unless the arrow is explicitly highlighted and free from ground suppression: Experiment 4), but that people who knew about the possibility of a hidden arrow became faster to respond to the target, regardless of cueing direction and congruency (Experiment 1–3). These results are consistent with the Biased Competition Model in figure-ground research, and highlights how basic research in perception can be readily applied to explain real-world applications. Our conclusion is twofold: (1) no, people do not unconsciously perceive the FedEx arrow, and (2) but knowing about the arrow can fundamentally change the way we visually process these negative-space logos in the future.

## Data Availability

The datasets used and analyzed during the current study are available from the corresponding author on reasonable request.

## Footnotes

1 In the “reverse control” images, although there is no arrow, the relocation of “x” nonetheless leaves a triangle (i.e., arrowhead) intact, pointing left or right (Fig. 1, reverse control images for left and right). To check whether these “semi-cues” would create a cueing effect, we reconducted a 5 × 2 ANOVA, where there are 5 levels of cue congruence (congruent vs. incongruent vs. neutral vs. semi-incongruent vs. semi-congruent). The results are the same as reported above [prior knowledge: F(1,125) = 5.431, p = 0.021, η^2^_p_ = 0.0042; cue congruence: F(4,500) = 1.303, p = 0.273, η^2^_p_ = 0.010; interaction: F(4,500) = 1.758, p = 0.157, η^2^_p_ = 0.014], where the ‘yes’ group is significantly faster than the ‘no’ group across all cue types.

2 We again performed a 5 × 2 ANOVA to check on the possible effect of the “semi-cues” from the reversed images. We found similar results with the main analysis [prior knowledge: F(1,45) = 3.734, p = 0.060, η^2^_p_ = 0.077; cue congruence: F(4,180) = 1.380, p = 0.252, η^2^_p_ = 0.030; interaction: F(4,180) = 1.323, p = 0.263, η^2^_p_ = 0.029].

3 In the present experiment, we did not remove participants that reported seeing the blue arrow in their post-experiment questionnaire. To test if this new exclusion criteria would significantly change our previous results, we reconducted the 5 × 2 ANOVA for Experiment 1 and 2 while including all participants who reported noticing the arrow, the results are the same as what we have reported in Experiment 1 [Prior knowledge: F(1,131) = 4.887, p = 0.029, η^2^_p_ = 0.036; cue congruence: F(2,262) = 1.862, p = 0.161, η^2^_p_ = 0.014; interaction: F(2,262) = 0.140, p = 0.852, η^2^_p_ = 0.001] and Experiment 2 [Prior knowledge: F(1,48) = 4.258, p = 0.044, η^2^_p_ = 0.081; cue congruence: F(2,96) = 1.035, p = 0.345, η^2^_p_ = 0.021; interaction: F(2,96) = 0.786, p = 0.431, η^2^_p_ = 0.016].

4 Conducting 5 × 2 ANOVA with semi-cues yielded similar results [prior knowledge: F(1,62) = 7.544, p = 0.008, η2_p_ = 0.108; cue congruence: F(4,248) = 0.573, p = 0.654, η^2^_p_ = 0.009; interaction: F(4,248) = 1.252, p = 0.292, η^2^_p_ = 0.020].

5 Rerunning the 5 × 2 ANOVA with semi-cues yielded similar results, where prior knowledge is significant [prior knowledge: F(1,206) = 3.892, p = 0.050, η^2^_p_ = 0.019] but others are not [cue congruence: F(4,824) = 1.876, p = 0.124, η^2^_p_ = 0.009; interaction: F(4,824) = 0.378, p = 0.808, η^2^_p_ = 0.002].

6 We also applied the same analysis for Experiment 4a by including participants who reported noticing the FedEx-like arrow during the experiment, the results were the same as originally reported [Prior knowledge: F(1,218) = 1.015, p = 0.315, η^2^_p_ = 0.005; cue congruence: F(2,436) = 1.016, p = 0.352, η^2^_p_ = 0.005; interaction: F(2,436) = 0.088, p = 0.886, η^2^_p_ < 0.001].

7 Reconducting the ANOVA to include the semi-cues would dilute all the effects such that both main effects and their interaction would not be significant [prior knowledge: F(1,106) = 0.013, p = 0.911, η2_p_ < 0.001; cue congruence: F(4,424) = 1.409, p = 0.247, η^2^_p_ = 0.013; interaction: F(4,424) = 1.336, p = 0.264, η^2^_p_ = 0.012].
